# Early intervention influences positively quality of life as reported by prematurely born children at age nine and their parents; a randomized clinical trial

**DOI:** 10.1186/s12955-015-0221-9

**Published:** 2015-02-22

**Authors:** Inger Pauline Landsem, Bjørn Helge Handegård, Stein Erik Ulvund, Per Ivar Kaaresen, John A Rønning

**Affiliations:** Child and Adolescent Department, University Hospital of Northern Norway, Tromsø, Norway; UiT, Health Faculty, The Arctic University of Norway, Tromsø, Norway; UiT, RKBU Nord, The Arctic University of Norway, Tromsø, Norway; Department of Education, University of Oslo, Oslo, Norway

**Keywords:** Preterm children, Early intervention, Quality of Life, Long-term follow-up

## Abstract

**Background:**

The Tromsø Intervention Study on Preterms evaluates an early, sensitizing intervention given to parents of prematurely born children (birth-weight < 2000 g). The current study investigated the potential influence of the intervention on children’s self-reported and parental proxy-reported quality of life (QoL) at children’s age of nine.

**Methods:**

Participants were randomized to either intervention (PI, n = 72) or preterm control (PC, n = 74) in the neonatal care unit, while healthy term-born infants were recruited to a term reference group (TR, n = 75). The intervention was a modified version of the Mother-Infant Transaction Program, and comprised eight one-hour sessions during the last week before discharge and four home visits at 1, 2, 4 and 12 weeks post-discharge. The two control groups received care in accordance with written guidelines drawn up at the hospital. Participants and parents reported QoL independently on the Kinder Lebensqualität Fragebogen (KINDL) questionnaire. Differences between groups were analyzed by SPSS; Linear Mixed Models and parent–child agreement were analyzed and compared by intra-class correlations within each group.

**Results:**

On average, children in all groups reported high levels of well-being. The PI children reported better physical well-being than the PC children (p = 0.002). In all other aspects of QoL both the PI and the PC children reported at similar levels as the term reference group. PI parents reported better emotional wellbeing (p = 0.05) and a higher level of contentment in school (p = 0.003) compared with PC parents. Parent–child agreement was significantly weaker in the PI group than in the PC group on dimensions such as emotional well-being and relationships with friends (p < 0.05). PI parents reported QoL similar to parents of terms on all aspects except the subscale self-esteem, while PC parents generally reported moderately lower QoL than TR parents.

**Conclusions:**

This early intervention appears to have generated long-lasting positive effects, improving perceived physical well-being among prematurely born children and parent’s perception of these children’s QoL in middle childhood.

**Trial registration:**

Clinical Trials Gov NCT00222456.

## Background

It is important to include measurement of health status and QoL in neonatal long-term follow-up studies, because interventions in the neonatal period may have effects that only become evident after a period of latency in toddlerhood [1]. Until recently, long-term developmental outcomes on prematurely born children have been dominated by reports of functional ability and the presence or absence of physical sequelae [1-5]. Perceived health and quality of life and physical and cognitive functioning are related but not identical concepts [1,6]. It has been shown that quality of life can be improved beyond symptom levels, thus psychopathology does not have a simple linear relationship to well-being [7]. Mental and social well-being is fundamentally important as reflected in the saying; “it is not how life is, but how the individual can deal with it that matters”.

The World Health Organization has defined Quality of life (QoL) as “an individual’s perception of their position in life in the context of the culture and value systems in which they live and in relation to their goals, expectations, standards and concerns” [8]. QoL is a holistic concept of well-being, even though the concept may be interpreted and described differently [6]. Descriptions of QoL frequently cover factors such as; a subjective phenomenon, a multidimensional construct and an aspect which is related to physical, psychological and social dimensions that includes both positive and negative facets of life [6,9,10]. Thus, it is not possible to directly observe QoL and no universal definition is available [9].

Studies reporting global or health-related aspects of QoL among preterm children are few. They consist mainly of parents’ proxy reports on pre-school children or self-reported QoL by adolescents or young adults [6,11]. These studies confirm that prematurity often implies a heavier developmental burden related to morbidity, use of extra health-care services, having fewer friendships, and lower level of education [11-13]. Although several studies have reported that the differences between preterm and term born children diminish with time [12-14], others conclude that being born with a low birth weight has long-lasting negative implications for mental health and quality of life as perceived by the individual concerned [15]. One single, small study has reported self-rated QoL among preterm children at middle school-age [16]. These children scored their health-related QoL significantly lower than term peers at age 9 to 10 years, in line with studies that reported parental proxy QoL in preterm children at this age [12,17].

The need for interventions which could strengthen the QoL of preterm children has been pointed out [15,18] but as far as we know, no results have yet been published. This study looked at whether early sensitization of parents of preterms (birth weight < 2000 g) could positively influence children’s and parent’s proxy perception of QoL in middle school-age. The sensitizing intervention program took place in the newborn period, and its primary focus was to reinforce the parent–child relationship [19]. Parents were introduced to their infant’s social availability; they were taught to identify their child’s signs of stress and how they could adjust their own activities and interactions to suit their child as well as possible [19,20]. This was intended to improve both parental confidence and parent–child co-regulation, offering more possibilities for mutual joyful and successful interactions.

In accordance with a transactional understanding of development, better co-regulation in these families was expected to enable them to adapt to new developmental challenges as the child grew [21]. Better co-regulation would confirm parental perception of their own role as good caregivers for their child and probably contribute to a more relaxed family climate. On the other hand, increased parenting stress has frequently been associated with less successful co-regulation [22,23] and has been described as influencing children’s quality of life from the earliest years [24,25]. Tu *et al.* [23] reported that maternal stress had an important modulating functioning for the preterm infant’s capacity to recover from early pain-related distress in infancy. They reported high levels of cortisol to be strongly associated with the preterm’s infant’s capacity to focus attention at eight months when exposed to high levels of maternal parenting stress. Lee *et al.* [25] described how QoL, as perceived by the primary caregivers, was directly related to parenting stress, which in turn was directly related to children’s proxy reported QoL at preschool age. A persistent reduction in parenting stress has already been reported in our study by mothers in the intervention group, compared to mothers of preterm controls [26,27]. These results are thought to influence children’s and parents’ reports of QoL at age nine and will be incorporated in the analyses. QoL has also been described as being powerfully influenced by emotional and behavioral problems, and prematurely born children have repeatedly been reported as having higher levels of attentional, social and internalizing difficulties than term born children [5]. In our study, better cognition and fewer behavioral problems were reported in the intervention group at pre-school ages [28,29]. These tendencies seem to persist throughout childhood as fewer attentional problems and better adaptation to school have been reported on the PI group until age nine [30].

On the basis of previous findings we hypothesized that children and parents in the intervention group would report better quality of life than the preterm control group. A definition by Jozefiak of an “inner QoL”, which addresses solely the subjective experiences of QoL, was modified to this study; “QoL is the subjective reported well-being in regard to the child’s physical and mental health, self-esteem, perceived relationship to friends and families as well as to school” [10,31]. An additional question raised in this study pays attention to the level of intra-familiar child and parental-proxy agreement. A previous review of QoL studies focusing on young children with various health-conditions reported this level of agreement to be affected by children’s age and health but also with great variability between studies [32]. The need of more studies was highlighted. Jozefiak et al. [10] reported significant but low correlations between parents and children’s reports in their school selected sample. Positive maternal perceptions of children’s emotional well-being have previous been reported to be negatively and significantly related to maternal involvement [33]. We wonder if the intervention may have changed the parent–child agreement concerning measures of QoL. We have already reported a more successful adaptation to school requirements among the PI children which may indicate that these children evaluate their quality of life more independently from their parents than the preterm controls [30]. This study asks three questions: Did the early intervention influence preterm children’s self-reported QoL and the parental proxy reports of QoL at age nine? Secondly, did the intervention affect the level of agreement between child and parental proxy reported QoL in the two preterm groups? Thirdly, was QoL reported by children and parents in each of the two preterm groups at similar levels as QoL reported by children and parents in the term reference group?

## Methods

### Participants

This study is part of a comprehensive clinical trial; the Tromsø Intervention Study on Preterms (TISP) which recruited infants with BW < 2000 g between March 1999 and September 2002 (Rønning JA, Ulvund SE, Dahl LB, Kaaresen PI: Study-protocol, 1998, unpublished). Computer-generated random numbers were use to allocate preterm infants to an intervention group (PI, n = 72) or a control group (PC, n = 74). The randomization was performed in blocks of 4 to 6 and was stratified according to gestational age (GA) < 28 and GA ≥ 28 weeks. Healthy newborns (GA ≥ 37 weeks and BW > 2800 g) were also recruited from the well-infant nursery to form a term reference group (TR, n = 75). Parents of the first baby born after a preterm infant allocated to the preterm intervention group were asked to participate in the study. If they declined the next family was asked. Study design and calculation of sample size have been described in detail in previous publications [26]. Written informed consent was received from all adult participants before inclusion. Preterm controls (PC) followed the NICU’s guidelines for discharge of preterm infants, while term references (TR) were routinely examined once by a pediatrician on their third day of life. Demographical baseline data for each study group have previously been described in detail [26], and are summarized in Table 1.

**Table 1:** **Birth, medical and demographic characteristics at randomization**

	**PI Group N = 72**	**PC Group N = 74**	**TR Group N = 75**
*Infant characteristics*			
BW, mean ± SD, g	1396 ± 429	1381 ± 436	3619 ± 490
400 - 1000 g, *n* (%)	20 (28)	20 (27)	
1001 - 1500 g, *n (*%)	15 (21)	20 (27)	
1501 - 2000 g, *n (*%)	37 (51)	34 (46)	
GA, mean ± SD, wk	30.2 ± 3.1	29.9 ± 3.5	39.3 ± 1.3
< 28 wk, *n (*%)	17 (24)	19 (27)	
28 - 32 wk, *n* (%)	36 (50)	37 (50)	
≥33 wk, *n* (%)	19 (26)	18 (24)	
Boy, *n* (%)	38 (53)	39 (53)	40 (54)
Twin, *n* (%)	16 (22)	14 (19)	0
SGA	11 (14)	10 (13)	
Prenatal steroid use, *n* (%)	53 (74)	57 (77)	
SNAP II, mean ± SD	8.3 ± 10.9	10.4 ± 11.3	
CRIB score, mean ± SD, *N* = 85	3.2 ± 2.8	2.7 ± 2.9	
Received ventilation, *n* (%)	29 (40)	37 (50)	
Duration of ventilation, *n* (%)	7.0 ± 18.6	7.1 ± 17.3	
Postnatal steroid use, *n* (%)	9 (13)	10 (14)	
Oxygen therapy at 36 wk GA, n (%)	11 (15)	14 (19)	
Abnormal cerebral ultrasound, *n* (%)			
IVH grade 1 or 2	7 (10)	8 (11)	
IVH grade 3 or 4	3 (4)	5 (7)	
Periventricular leukomalacia	4 (6)	8 (11)	
*Maternal and social characteristics*			
Mother’s age, mean ± SD, y	30.8 ± 6.1	29.1 ± 6.4	29.7 ± 6.1
Firstborn child, *n* (%)	40 (56)	37 (54)	27 (37)
Mother’s education, mean ± SD,^a)^	14.6 ± 2.8	13.5 ± 3.2	14.9 ± 2.8
Father’s education, mean ± SD,^a)^	13.8 ± 3.1	13.5 ± 3.2	14.4 ± 3.2
Mother’s monthly income,^b)^	15.8 ± 7.7	14.6 ± 6.7	15.9 ± 8.0
Father’s monthly income,^b)^	21.1 ± 8.7	19.9 ± 8.1	21.9 ± 9.8

^a)^ = education in years.

^b)^ = in Norwegian 1000 kroner, calculated for 131 families due to 15 twins.

CRIB = Clinical Risk Index for Babies.

IVH = Intraventricular Hemorrhage.

SGA = defined as BW > 2SD below the mean for GA.

SNAP II = Score of Acute Neonatal Physiology II.

### Intervention

The intervention program was a modified version of the Mother-Infant Transaction Program (MITP), a further development of the Neonatal Behavioral and Assessment Scale (NBAS) [18,34]. MITP is designed as a stepwise parental guidance process, with gradually increasing complexity in the knowledge offered to parents [19]. Each family received eight one-hour sessions during the final week before discharge from hospital, and four home visits at 1, 2, 4, and 12 weeks post-discharge [19]. The modification of the MITP included an initial session during which parents could vent feelings such as grief, anger or frustration related to the preterm delivery, the hospital stay and how those conditions had affected their life (Study-protocol, unpublished). The MITP aimed to 1) enhance parents’ understanding of their child’s expressions, and 2) promote sensitive, positive and practical transactions between parents and child. Eight nurses were trained to perform the intervention and each family was guided by the same nurse during all the sessions.

All of the mothers participated in all the sessions, while the fathers’ mean participation rate was 6.5 sessions (SD = 3.4), which constituted 54% of the intervention program. At first, the parents and the interventionist investigated the child’s capacities, focusing on the baby’s readiness and social communication abilities. During the following sessions, the parents were helped to recognize and be sensitive to behavioral cues, signs of disturbed regulation, and stress in the child’s physiological, motor and state organization. In the last two sessions before discharge this knowledge was combined with daily caring activities such as bathing, feeding and preparation for sleep. Parents were helped to make adjustments to their child’s strengths and vulnerabilities, resulting in reduced levels of stress and maximizing their social engagement with their babies. During the four home visits, these topics were revisited and fine-tuned to individual needs, especially in connection with the child’s temperament, which was one of the main topics of the third home visit. The families had no further contact with the interventionists and in contrast to the original MITP study, parents did not receive a logbook of the interventions [19]. Consistent implementation of the intervention was ensured by a review of logbooks carried out by the study director (JAR).

### Follow-up procedures

All participants received the same medical, developmental, and psycho-social assessments on all follow-ups. Recommendations about contacting other services (physiotherapy, pedagogical-psychological services, child habilitation, specialized child psychiatric services and child welfare authorities) were given if needed throughout childhood (age 6 months, 1, 2, 3, 5, 7, 9 years). TISP was approved by the Regional Committee for Medical Ethics and the Norwegian Data Inspectorate on three occasions (in 1999, 2005 and 2010).

### Data collection

Approximately 14 days before the nine-year follow-up session questionnaires were sent to the families [31]. Parents and children were requested to report QoL independently.

### Measures

#### Child and parent-reported quality of life

The KINDL-questionnaires consist of a self-report questionnaire (Kid KINDL®) appropriate for children (7 to 13 years), and a questionnaire for parental proxy report (KINDL® for parents) [31,35]. These questionnaires are short, generic and have been translated for use in Norwegian populations [9,31]. Each comprises 24 corresponding items that are equally formed as either positive or negative statements about different facets of the child’s life. Each item addresses experiences over the past week and is rated on a five-point scale; ^1)^never, ^2)^seldom, ^3)^sometimes, ^4)^often and ^5)^always. Outcomes consist of a global QoL sum-score and six subscales; physical well-being, emotional well-being, self-esteem, family, friends and school. Mean scores are calculated for each of the subscales and total score and linearly transformed to a 0 to 100 scale, on which higher scores indicate better QoL. The questionnaire was validated by Jozefiak et al. [9]. Relatively low internal consistency (Cronbachs alpha) were reported by the 4th grade students (9 – 10 years) on the subscales; emotional well-being (0.52); friends (0.49) and school (0.47) but fairly acceptable reliability on the others; (total scale (0.83); physical well-being (0.66); family (0.62) and self-esteem (0.68). All versions of the KINDL questionnaire are supplemented with a “disease-module” consisting of a filter question and six items about possible long-lasting illness or current hospital admission.

#### Parenting stress index

Mothers and fathers reported via the Parenting Stress Index (PSI) full version on all follow-ups until seven years of age and correspondingly on the PSI short version (PSI-SF) at age nine [27].

#### Children’s behavior

Children’s behavior problems were reported on the Child Behavior Checklist (CBCL) [30] at ages 2, 3, 5, 7 and 9. At ages 7 and 9 teachers reported on Teacher Report Form (TRF) [30].

#### Demographic, birth and medical factors

Birth and medical information was collected from medical records at inclusion time. Socio-demographic variables were reported by parents before discharge from the hospital (Table 1.).

### Analysis

Previous studies have shown that the intervention has an effect on child and parent-related stress and child behavior [26-30]. At nine years, stress and behavior are correlated with QoL variables and therefore used as covariates in analyses that tested group differences on QoL measures. Because of the clustering effects of twin pairs, groups were compared by means of multilevel modeling (Linear mixed models (LMM), SPSS statistics, version 20) [36]. Analyses were controlled for birth and medical factors and those that influenced outcome measures were included in the analyses to increase the validity of group comparisons. Agreement between parent’s and children’s scores in the different study groups was analyzed by intraclass correlations (ICC), and the difference between the two independent intraclass correlation coefficients for the PI and the PC groups was tested as described by Alsawalmeh & Feldt [37]. Effect sizes (ES) created by the use of Hedges’ g are reported on predicted differences in mean scores between groups [38]. An effect size below 0.40 is usually regarded as small, a value between 0.40 and 0.60 as moderate and finally viewed as strong if ES exceeds 0.60. A p-value < 0.05 was considered significant.

## Results

### Participation and comparisons of background variables

**Figure 1:**
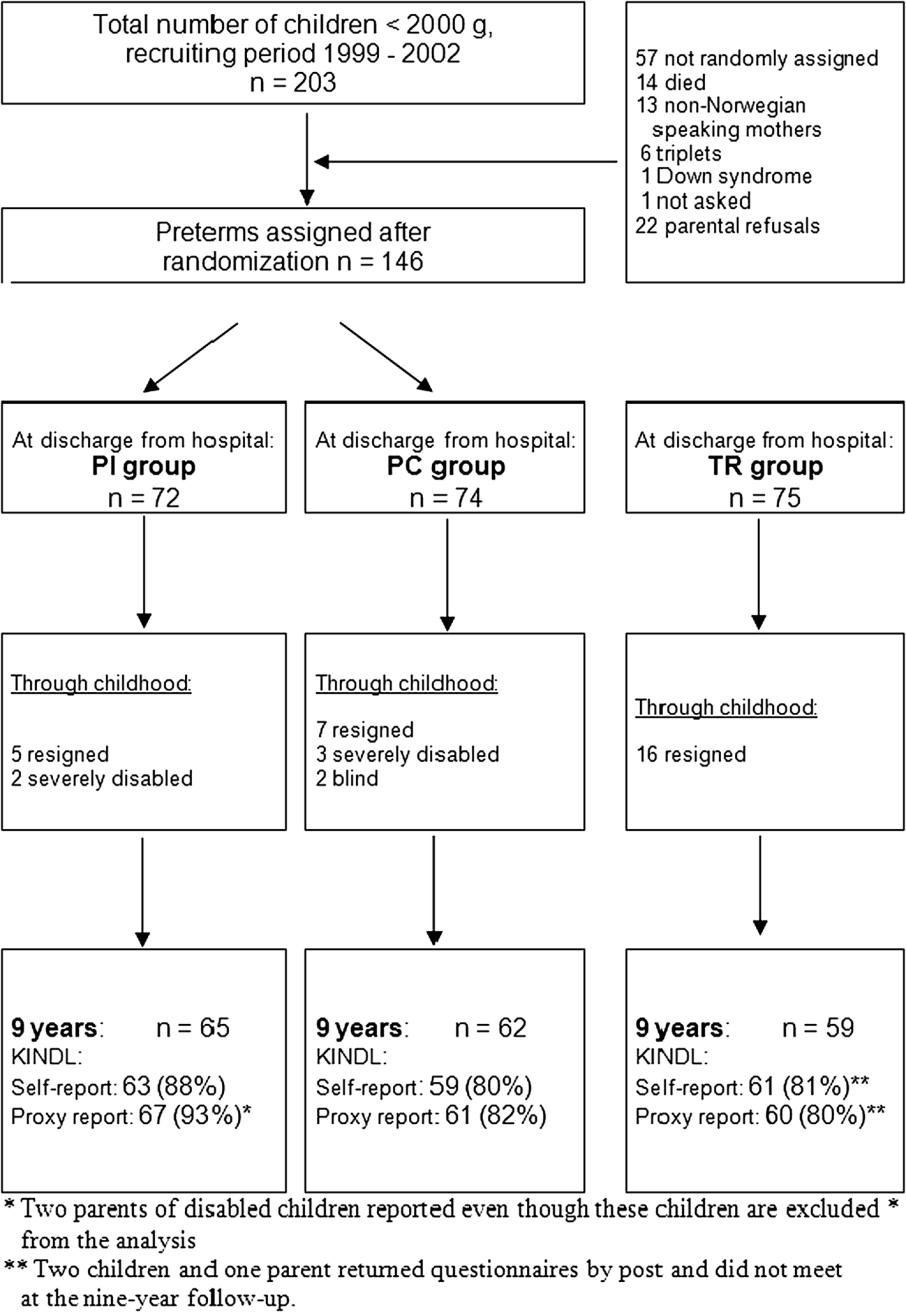
**Study flow.**

Randomization resulted in well-balanced preterm groups with one exception. There was a significant difference between the preterm groups in terms of maternal education, as the PI mothers had an average of one year more of education than the PC group at the time of inclusion in the study (Table 1). However, maternal education had no influence on group comparisons in this study. Participation rates remained very high throughout the study. At nine years, the response rates on QoL across groups were 83% on children’s self-reports and 85% on parental proxy reports (Figure 1). Fewer children were lost to follow-up in the PI group compared to the PC- and TR group. PC children, who did not respond to the Kindl questionnaire tended to be reported with more neonatal morbidity (SNAP II and Oxygen at 36 weeks GA) compared to PI children who dropped out, even though no statistical differences appeared. Mothers were the main informant of QoL proxy reports in all groups (PC: 84%, PI: 74%, TR: 92%).

### Self-reported QoL in the PI and the PC group

PI children reported significantly higher QoL than PC children on the subscale “physical well-being” (F (1, 103) = 10.2, *p* = 0.002, ES = 0.57) controlling for birth-weight (BW) and neonatal illness severity (SNAP II). Physical well-being reported by children was influenced by BW (F (1, 118) = 6.5, *p* = 0.012) and SNAP II (F (1, 118) = 6.1, *p* = 0.015), indicating that children with lower BW or more severe neonatal illness tend to report physical well-being somewhat lower in both preterm groups. Children’s physical well-being at age 9 was not influenced by children’s gender but significantly associated with maternal (F (1, 118) = 7.6, p = 0.007), paternal (F (1, 97) = 5.7, p = 0.018) and teacher (F (1, 104) = 8.6, p = 0.004) report of total behavior problems at age 7. Finally, parents’ proxy reports of physical well-being were strongly associated with children’s reports (F (1, 108) = 36.0, *p* < 0.0005) but in that case the impact of BW and SNAP became non-significant, while the difference between the PI and the PC group endured (F (1, 104) = 8.4, *p* = 0.005). No significant differences between the PI and the PC group were found in self-reported quality of life on global QoL or the other subscales.

### Parental proxy reported QoL in the PI and the PC group

PI parents reported significantly higher QoL than PC parents on two KINDL subscales. The first difference appeared in the subscale “emotional well-being” (F (1, 112) = 3.9, *p* = 0.05, ES = 0.34) when BW, SGA and SNAP II were controlled for; all of these were significantly associated with this outcome. Parental reports of emotional well-being were strongly associated with maternal report of child-related stress at age 7 (F (1,116) = 56.1, p < 0.0005). Similar associations were revealed between stress reported at age 2, 3 and 5 and emotional well-being, all of which made the impact of group allocation non-significant. Next, PI parents reported higher QoL on the subscale “school” (F (1, 116) = 9.2, *p* = 0.003, ES = 0.54) than PC parents after controlling for BW, SGA and SNAP II. Male gender was associated with lowered QoL in the school dimension (F (1, 115) = 8.1, *p* = 0.005) but this association disappeared when the significant association with teacher’s report of attentional problems at 9 years had been controlled for (F (1, 99) = 32.7, *p* < 0.0005). A trend towards a difference between the PI and the PC group was found on parental reports of Total QoL before controlling for birth and medical factors (F (1, 113) = 4.0, *p* = 0.054, ES = 0.32).

**Figure 2:**
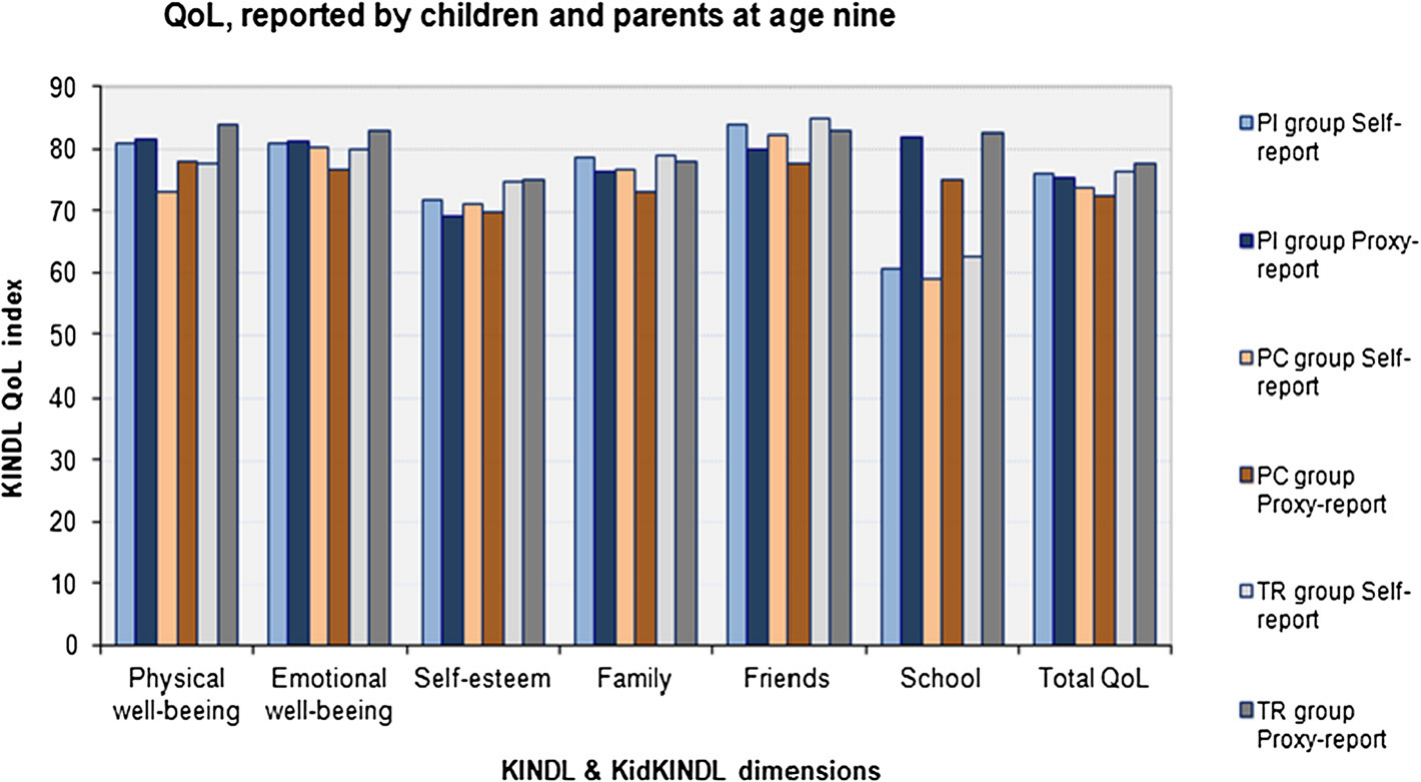
**Mean QoL reported at 9 years by children and parents in the PI-, PC- and TR group.**

Means of all QoL outcomes as reported by children and parents are presented in Figure 2. The strength of group comparisons are reported Table 2.

**Table 2:** **Strength of significant differences between study-groups (ES)**

	**PI scores > PC**		**TR scores > PC**		**TR scores > PI**	
	**Children**	**Parents**	**Children**	**Parents**	**Children**	**Parents**
**Physical well-being**	0.57**	-	-	0.44*	-	-
**Emotional well-being**	-	0.34*	-	0.52**	-	-
**Self-esteem**	-	-	-	0.46*	-	0.37*
**Family**	-	-	-	0.46*	-	-
**Friends**	-	-	-	0.46*	-	-
**School**	-	0.54**	0.32 (*)	0.55**	-	-
**Total QoL**	-	0.34(*)	-	0.65***	-	-

Effect size (ES) = Hedges’ g.

Level of significance: (*) = p <0.08; * = p < 0.05; ** = p < 0.01; *** = p < 0.001.

### Agreement between children’s and proxy-reported QoL

Intraclass correlations between children’s and parent’s reports of QoL varied between the KINDL-subscales and to some degree between groups (Table 3).

**Table 3:** **Parent–child agreement in the three study groups and across all groups**

**KINDL®**	**PI group**	**PC group**	**TR-group**	**Across groups**
	**(n) ICC**	**(n) ICC**	**(n) ICC**	**(n) ICC**
**Physical well-being**	(62) 0.57	(59) 0.48	(60) 0.46	(181) 0.50
Emotional well-being*	(63) 0.19	(59) 0.50	(60) 0.41	(182) 0.36
**Self esteem**	(63) 0.34	(58) 0.49	(60) 0.42	(181) 0.43
**Family**	(63) 0.53	(59) 0.37	(60) 0.53	(182) 0.49
Friends* ^**,**^ **	(63) 0.31	(59) 0.60	(60) 0.38	(182) 0.46
**School**	(61) 0.22	(58) 0.21	(60) 0.04	(179) 0.17
**Total QoL**	(63) 0.67	(59) 0.57	(60) 0.51	(182) 0.60

ICC: Intraclass-correlation.

n: number of parent–child pairs.

*Significant difference in parent–child agreement between the PI and the PC group (p < 0.05).

**Significant difference in parent–child agreement between the PC and the TR group (p < 0.05).

Significant differences between the PI and the PC group were detected in the subscales “emotional well-being” and “friends”. In both cases the agreement between parents and children in the PI were low compared to the PC group. A similar difference between the PC and the TR group was detected on the subscale “friends”, while no significant differences were revealed between the PI and the TR groups.

### Reports of QoL in the PI and the PC groups compared with the term reference group

#### The PC group compared with the TR group

Children in the PC group reported QoL at the statistically same level as term references, even though they tended to report lower QoL, especially on the subscale “school” (F (1, 119) = 3.2, p = 0.08, ES = 0.32) (Figure 2). On the other hand, parents in the PC group reported consistently lower QoL compared to TR parents on all subscales which constituted a five-point difference in mean Total QoL (F (1, 114) = 11.7, *p* = 0.001, ES = 0.65) Table 2.

#### The PI group compared with the TR group

Children in the PI group reported QoL similar to the TR group on all outcomes. The same pattern emerged in the parental proxy reports, with one exception. PI parents perceived their children as having less self-esteem than did parents in the TR group (F (1, 119) = 6.5, *p* = 0.012, ES = 0.37). This difference disappeared when controlling for children’s birth weight and paternal income at inclusion time, as lowered self-esteem was related to lower birth-weights and lower paternal income.

## Discussion

This is the first paper from TISP in which the children themselves have reported outcomes independently of their parents. Previous reports of behavioral, motor and cognitive outcomes throughout childhood have indicated several positive effects of the intervention program [26-30,39-42]. This is now supplemented by reports of QoL, and the PI children differ from the PC children, as they experienced significantly higher physical well-being (subsequently named bodily well-being) at age nine, while PI parents perceived significantly higher emotional well-being and a better school-related life among their children compared to parents of preterm controls. Our hypothesis is supported as the intervention may generate a better quality of life among preterm born children and we suggest that the early intervention can have long-lasting positive effects on well-being in families rearing prematurely born children.

In general, QoL was reported at relatively high levels across all groups. Mean scores were mostly above 75 on total QoL and subscales (except school-related QoL) and were comparable to the general population of Norwegian children aged 8 to 16 years reported by Jozefiak [9,10]. Studies reporting QoL among preterm children of middle school-age are few, and those published have employed different definitions and measurements [6,16,17]. Self-reported QoL by preterm children at this age seems to have been only reported once [16], and to the best of our knowledge, this is the first report on QoL as an outcome in a RCT of an early intervention program in preterm children. Comparisons of results with other studies of QoL are therefore limited.

The PI children reported a higher level of bodily well-being than the PC group. Even though low BW and neonatal illness were negatively associated with QoL neither these nor other birth, medical or socio-demographic factors explained the group difference.

The difference was revealed by four questions that asked about children’s feelings of being strong and full of energy, tired or worn-out or suffering from illness, and headache or stomach-ache [31]. We assume these questions reflect an inner quality of life experience which may be subtle and not readily observable by parents. Both PI and PC parents reported their children as being at similar levels, and neither group reported differences in the disease dimension of the KINDL questionnaire. This fairly robust difference between the PI and the PC children may be an effect of the intervention. Olafsen *et al*. [40] reported a possible positive effect of the intervention on infant-parent co-regulation. They suggested that the intervention improved the PI mothers’ sensitization to their children’s regulatory competence across the first year because mother-infant dyads in the PI group had established a kind of co-regulation at age one, while a strong correlation between parental stress and children’s negative reactivity continued to be evident in the PC group. PI children were also reported by their parents to be more socially available at age one [41]. The development of infant self-regulation is a main developmental task in toddlerhood and preterms are particularly dependent on their parent’s ability to support their early immature regulatory efforts [43]. Feldman has described noteworthy coherence in regulatory patterns across early childhood, including the physiological regulation of cardiac vagal tone and sleep-awake cyclicity (becoming measurable in the last trimester of the pregnancy) and regulation of emotional, attentional and behavioral development until age five [44-46]. Early emotional regulation, and especially negative emotionality, was similarly found to predict several psychosomatic problems in middle childhood in a Swedish longitudinal study from age 11 months until 9 years [47]. The main associations were found in symptoms of headache and stomach ache both of which were also influenced by parental perception of parental control. In another Swedish study of 10-year-old school children [48], Svedberg *et al.* reported that 27% to 50% of the variance in QoL could be explained by psychosomatic symptoms. Problems frequently reported were sleeping problems, depression, problems of concentration and stomach-aches. These studies refer to aspects of child well-being that are closely related to the questions asked and findings reported above. We wonder if better bodily well-being in the PI group is caused by better, early parent–child emotional co-regulation and as such creates a more nourishing family climate with less stress, as has previously been reported [27].

On the other hand, PI parents perceived their children to have a higher QoL than the PC group in the dimensions emotional well-being and the child’s thriving in school. These analysis draw attention to the parental reports of stress, because parental proxy reports of emotional well-being were highly associated with maternal reports of child-related stress throughout childhood. The difference in emotional well-being between preterm groups seems to be fully explained by differences in maternal reports of stress. We have recently reported that PI mothers experienced less child- and parent related stress than PC mothers at all follow-ups until age nine. This consisted of statistically different patterns were PI children’s adaptability increased and moodiness decreased with age while PC children were reported at less preferable levels throughout childhood. Furthermore, PC mothers reported significantly more stress related to mother-child interactions at age nine [27]. The impact of parenting stress on children’s quality of life seems to largely agree with Lee *et al.* [25], who found that parenting stress was directly related to children’s QoL in both term and preterm populations. It is also in accordance with the findings of Østberg and Hagekull, who reported general parenting stress to be the primary predictor of maternal ratings of children’s adjustments [49]. Like Renk [33], these authors emphasize the importance of interventions that are able to change parental perception of children’s adjustments in a positive, accepting direction.

The second difference appeared in parental ratings of children’s well-being in school, where PI parents rated their children as enjoying a significantly higher QoL. Fewer attentional problems and more competencies in several aspects have previously been reported by parents and teachers in the PI group than the PC group [30]. It is not surprising that the same differences appear in parents’ reports of school-related QoL. Being able to stay focused and take in messages are essential skills for all children, enabling them to experience well-being, social belonging, and learning in school.

Preterm children in both groups rated their school-related QoL much lower than their parents did, a pattern similar to that previously described in population studies [9]. Children compare themselves with classmates every day and thus have more information about their strengths and weaknesses than their parents have. They may also be less aware of the period of time on which they were to report (only the previous week).

Concerning the second question asked, some differences in parent–child agreement did become visible. PI parents answered less similarly than their children (lower ICC) compared with the PC parents on the subscales “emotional well-being” and “friends”. (A similar difference was detected between the PC and the TR group on the subscale “friends”, with lower ICC in the term reference group). Less agreement between the parent–child reports may be perceived as less parental involvement in the children’s inner life. In the study of relationships between maternal perceptions and young children’s behavioral problems, Renk [33] showed that positive maternal perception correlated negatively and significantly with their involvement with their children, while the opposite pattern was described for negative maternal perception. Marques *et al.* [50] reported a higher QoL agreement between children diagnosed with Attention Deficit Hyperactivity Disorder (ADHD) and their parents than in typically developing children, which may support the view described above. On the other hand, QoL agreement between children and their parents was recently reviewed by Jardine *et al*. [32], and several factors appear to influence the levels of agreement (type of measurement, children’s age, parenting stress and the statistics employed). While higher inter-parental agreement concerning child and parent-related stress is supposed to indicate a well-functioning home environment [51] an opposite function may be related to the QoL agreement between parent and child, all of which makes the interpretation of results more difficult. Nevertheless, we suggest that the poorer agreement in the PI group is a sign of less involvement, due to less parental concern regarding the children.

A secondary finding was that both term and preterm children reported that their QoL was at a similar or lower level than their parents did. In a comparative study of QoL, Jozefiak *et al.* found that within the school sample, parents tended to rate their children’s QoL higher than the children did, while the opposite divergence of views was identified in the referred sample of children [52]. This was only assessed at a group mean level, but the overall impression is that both preterm groups largely show a similar pattern to that of the school sample referred to above.

No differences in self-reported QoL were evident between the preterm groups and the full-term references. The PC group tended to score below the level of the PI and the TR group on several scales. Because the focus of the KINDL questionnaire differs in several respects from other studies that report QoL in middle childhood [16], comparisons with other studies are uncertain.

The burden of prematurity became more visible in parental reports. PC parents consistently reported their children’s QoL as being lower on all subscales than did parents of terms. This is in agreement with previous reports [17], but somewhat surprising in view of the extensive follow-up program that offered continued opportunities to ask for help. Previous studies have identified parents of preterms as frequently experiencing a lack of professional support throughout childhood [17,53], and have suggested that this influences parents’ reports of QoL. Even though all families in the current study could potentially have benefitted equally from the follow-up program, and enjoyed equality of support in their search for other services (psychological, pedagogical, physiotherapy) [30], significant differences persisted.

On the other hand, PI parents reported QoL similar to the reports by parents of terms on all scales, except for slightly lower self-esteem. This is promising, as it suggests that the intervention had long lasting effects that almost normalized PI parents’ perceptions of their children in middle childhood. The KINDL self-esteem dimension includes statements such as; feeling proud of and pleased with oneself; having lots of ideas and being “on the top of the world” [10,31]. Preterm children have repeatedly been described as being more withdrawn and reticent than their full-term peers [2,5]. Such behavioral styles may have influenced parental proxy reports of self-esteem, as they may be perceived as signs of lowered self-esteem. Both groups of preterm children reported self-esteem at a similar level as term peers.

### Strengths and limitations

A major strength in this study is the RCT design and the high participation rate throughout the study. 83% of the children and 85% of parents reported on KINDL at nine years. However, several limitations need to be mentioned. First, a limitation is inherent in the nature of the self-reported questionnaires, in that parents and their child may have influenced each other’s reports. Families were requested to respond independently, but this could not be controlled as the questionnaires were completed before the follow-up session. On the other hand, the combination of self- and proxy reports is a strength as the two cover different aspects of children’s life [54]. Secondly, we need to address the limitations of self-reporting by nine-year-old children. The KINDL questionnaires were validated in a Norwegian cross-sectional survey [10]. Psychometric properties varied due to the children’s age, and internal consistency was lower than in the original German version of KINDL among the youngest children (age 8 to 10 years), especially on the subscales “friends”, “school” and “emotional well-being” [9,31]. On the other hand, Varni *et al.* concluded that self-reported health-related QoL may be reported by children as young as five [54]. Thirdly, comparisons with other studies are limited: ^1)^Different questionnaires cover different aspects of QoL making comparisons irrelevant, ^2)^Previous studies have reported on relatively old samples from the 1970s and 1980s and ^3)^most studies have tended to focus on extremely preterm children, who may have experienced more difficulties overall than our sample.

## Conclusions

This early intervention appears to have a long lasting influence on parental perceptions of their preterm child. First, PI parents reported that their children had significantly better “inner-QoL” on aspects of emotional wellbeing and contentment in school than parents in the PC group. Secondly, they reported a lower degree of parent–child agreement, which may indicate fewer parental concerns related to emotional and social functioning in the PI group. PI children reported better bodily well-being than the PC children. On all aspects of QoL except self-esteem, they are regarded by their parents as being similar to term peers. On the other hand, the parents of the preterm controls reported their children as having lower QoL in all areas (physical well-being, emotional well-being, self-esteem, family, friends and school) than did parents of terms at age nine.

## Abbreviations

BW: Birth weight
CBCL: Child behavior checklist
CI: Confidence interval
ES: Effect size
ICC: Intraclass correlation
KINDL: Kinder Lebensqualität Fragebogen
LMM: Linear mixed model
MITP: Mother-Infant Transaction Program
NBAS: Neonatal behavioral and assessment scale
NICU: Neonatal intensive care unit
PC group: Preterm control group
PI group: Preterm intervention group
PSI: Parenting stress index, full version
PSI-SF: Parenting stress index, short form
QoL: Quality of life
SGA: Small for gestational age
SNAP-II: Score for neonatal acute physiology
TISP: Tromsø intervention study on preterms
TR group: Term reference group
TRF: Teacher report form
